# An enhanced machine learning algorithm for type 2 diabetes prognosis with a detailed examination of Key correlates

**DOI:** 10.1038/s41598-024-75898-w

**Published:** 2024-11-01

**Authors:** Xueyan Wang, Ping Shen, Guoxu Zhao, Jiahang Li, Yanfei Zhu, Ying Li, Hongna Xu, Jiaqi Liu, Rongjun Cui

**Affiliations:** 1 Mudanjiang Medical University, Mudanjiang, China; 2 Shihezi University, Shihezi, China

**Keywords:** Feature selection, Hyperparametric optimization, Machine learning, SHAP, Significant correlates, Biological techniques, Computational biology and bioinformatics, Medical research

## Abstract

This study aimed to construct a high-performance prediction and diagnosis model for type 2 diabetic retinopathy (DR) and identify key correlates of DR. This study utilized a cross-sectional dataset of 3,000 patients from the People’s Liberation Army General Hospital in 2021. Logistic regression was used as the baseline model to compare the prediction performance of the machine learning model and the related factors. The recursive feature elimination cross-validation (RFECV) algorithm was used to select features. Four machine learning models, support vector machine (SVM), decision tree (DT), random forest (RF), and gradient boost decision tree (GBDT), were developed to predict DR. The models were optimized using grid search to determine hyperparameters, and the model with superior performance was selected. Shapley-additive explanations (SHAP) were used to analyze the important correlation factors of DR. Among the four machine learning models, the optimal model was GBDT, with predicted accuracy, precision, recall, F1-measure, and AUC values of 0.7883, 0.8299, 0.7539, 0.7901, and 0.8672, respectively. Six key correlates of DR were identified, including rapid micronutrient protein/creatinine measurement, 24-h micronutrient protein, fasting C-peptide, glycosylated hemoglobin, blood urea, and creatinine. The logistic model had 27 risk factors, with an AUC value of 0.8341. A superior prediction model was constructed that identified easily explainable key factors. The number of correlation factors was significantly lower compared to traditional statistical methods, leading to a more accurate prediction performance than the latter.

**Supplementary Information:**

The online version contains supplementary material available at 10.1038/s41598-024-75898-w.

## Introduction

The global prevalence of diabetes is increasing rapidly, with estimates suggesting that by the year 2045, around 48% of the world’s population will be affected by the disease^1^. Predominantly, over 90% of these cases will have type 2 diabetes^2^. Retinopathy, a prevalent and severe complication of diabetes, is currently the leading cause of blindness worldwide and significantly affects the quality of life of patients^3^. Research indicates that retinopathy occurs in 20%‒45% of diabetic cases^4^. In China, the overall prevalence of diabetes reached 14.92% between 2015 and 2021, with retinopathy complicating 22.4% of these cases^5^.

Scientific research consistently validates that identifying the key factors contributing to the development of diabetic retinopathy (DR) in patients with type 2 diabetes is a pivotal research focus, facilitating early prevention and treatment. Historically, research on DR prediction focused on image analysis^6^, the screening of DR risk factors using chi-square tests and t-tests, and the development of logistic regression analysis models. However, given the large sample size of type 2 diabetes clinical data, numerous influencing factors, and the risk of uneven sample distribution due to missing and redundant information, there are limitations to relying solely on the use of traditional statistical methods. For instance, it may lead to underfitting, diminished accuracy, and ineffective management of numerous features or variables^7,8^. Recently, machine learning algorithms have been extensively applied to assist clinicians in swiftly and accurately diagnosing and predicting DR ^9,10^. These algorithms primarily comprise traditional machine learning models (CML) and neural network (NN) based algorithms^11^. Despite the extensive research into machine learning algorithms for predicting DR and screening relevant factors, there remains ample scope for enhancing feature selection methods, identifying hyperparameters to improve model performance, and analyzing the significant impact of each feature on the outcome along with complex interactions between features.

In this study, we presented a concept for constructing, interpreting, and comparing machine learning models for DR prediction and the screening of significant correlates for clinical DR prevention and treatment.

This study was approved by the Medical Ethics Committee of Mudanjiang Medical University with the approval number 2022-MYSZR15 before it was conducted. The study followed all relevant ethical guidelines and regulations to ensure the rights and safety of participants.

## Methods

**Fig. 1:**
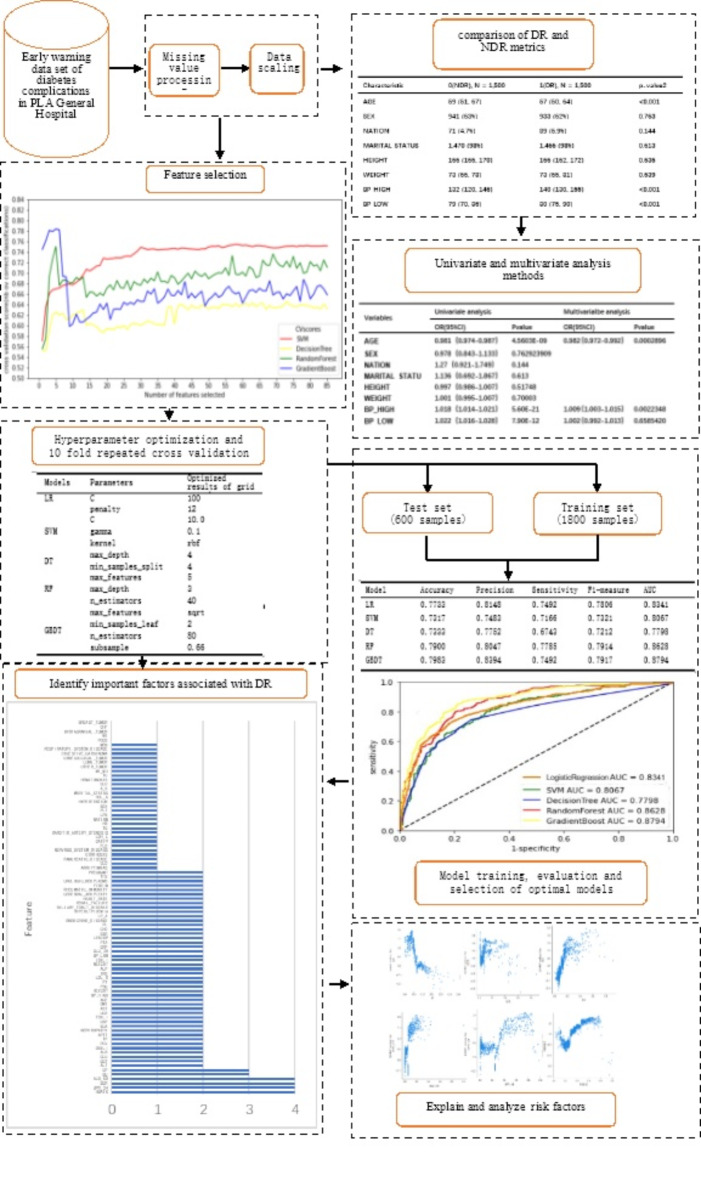
The research work process.

Our study comprised four phases: data pre-processing, model construction, significant correlated feature screening, model interpretation, and the comparison between traditional statistical methods and machine learning models. This study aimed to investigate factors influencing the accuracy of DR diagnosis and screening for significant risk factors. These factors can be outlined as follows: (1) identifying whether all attributes can act as significant predictive features; (2) determining if there are more suitable machine learning models; (3) verifying the importance of correlates screened by the model as crucial risk factors with DR predictive and diagnostic value; (4) examining how these significant risk factors affect the predictive outcomes of models; (5) understanding the benefits of machine learning models over traditional statistical models. Figure 1 graphically illustrates this concept of the study.

### Data sources

We utilized the "Diabetes Complication Early Warning Dataset of the General Hospital of the Chinese People’s Liberation Army," which was published by China’s National Population Health Science Data Centre. The study includes 3,000 diabetic patients, divided evenly into 1,500 cases with DR complications and 1,500 cases without such complications. This dataset contains 86 variables, as indicated in Supplementary Table 1.

### Data pre-processing

(1) Handling Missing Values: In the dataset, 52 of the 86 features had missing values. Thirteen features, including HEART_RATE (Heart rate), (GLU_2H) 2-h postprandial blood glucose, UPR_24 (Twenty-Four Hours Urinary Protein), UCR (Urine creatinine), CP (C-peptide), INS (Insulin), ESR (Erythrocyte sedimentation rate), LP_A (Lipoprotein-A), PL (Phospholipid), FIBRIN (Fibrin), M1_M2 (M1 macrophages and M2 macrophages), TH2 (T helper 2 cell), and BUN (Blood urea nitrogen), exhibited missing values exceeding 50%. However, except for BUN, all other features were retained due to their significant impact on the study. Upon observing these 12 retained features, the prevalence of DR was determined to be 50.3%, 66.8%, 75.9%, 76.5%, 72.7%, 68.9%, 60.7%, 20.2%, 54.3%, 30.4%, 25%, 35.7%, and 0%. Consequently, BUN was excluded, leaving the remaining 12 features that impacted the study intact.

Categorical feature values were filled with the mode, and continuous eigenvalues were filled with the mean^12,13^. There were 51 continuous features in the dataset, of which 48 features had missing values, and the number of missing values for 30 features accounted for less than 10% of the overall data. The "Blood urea nitrogen" column was empty, so we deleted it. The number of missing values for the remaining 17 consecutive features accounted for more than 10% of the overall number, and the mean filling method was also used. In subsequent studies, we considered using more complex data imputation methods (such as multiple imputation, model-based methods, etc.) to further improve the data processing. There are 35 category features in this dataset, among which only 1 feature has missing value, and the number of missing values accounts for 0.67% of the total sample number. We used the mode to fill the missing value, which can better reflect the central trend of the data.

(2) Data Normalization: In the Python 3.7.0 programming environment, we utilized Scikit-learn’s Min-Max Scalar to scale the continuous-type feature variables, resulting in data values within the [0,1] interval. This normalization process unified the dimensions of each feature, improved the performance of the model, accelerated the calculation speed, and enhanced the interpretability of the model. This method enabled us to mine the valuable information in the data more efficiently and accurately in the subsequent data analysis and model training process.

### Statistical perspective

We used Statistical Package for Social Sciences (version 26.0) for statistical analysis. When comparing the indicators between the two groups of patients, we expressed counting data as percentages (%). The quantitative data were described as "mean ± standard deviation" in the case of a normal distribution and as "median (interquartile spacing)" for a non-normal distribution. Logistic analysis was used to correlate factors following the idea of "single first, then multiple," with *p* < 0.05 deemed statistically significant. The logistic classification model was used to evaluate R4.2.2. The statistically significant variables in the univariable logistic regression analysis were first included in the logistic regression model to derive the correlates of DR and the AUC values of the model.

### Machine learning model and feature selection

In this study, we employed SVM, DT, RF, and GBDT machine learning algorithms, which are commonly used in diabetes-related research for binary classification problems, to construct the model^14–17^. The recursive feature elimination cross-validation (RFECV) algorithm was used to select features from the pre-processed dataset. RFECV underwent multiple training cycles, each time removing the least important features from the current feature set. The algorithm validated the performance of different feature combinations through cross-validation. RFECV directly optimizes for a given model, typically identifying a feature subset with superior classification performance and better model generalization capabilities.

### Partitioning of training, testing, and external validation sets

To enhance the ability of the four machine learning models to generalize and prevent overfitting, the dataset was divided into three parts: a training set, a verification set, and a test set. Typically, 50% of the samples were used as a training set to fit the model, while 25% of the samples were used as a verification set to estimate the prediction error and provide a basis for model selection. The remaining 25% of the test set was used to assess the generalization error of the final selected model.

### Machine learning model optimization

In this investigation, we determined the optimal values for the machine learning algorithm by tuning the hyperparameters of the model. This approach provided superior performance compared to using default parameters. To reduce memory overhead and runtime, we only tuned hyperparameters that substantially contributed to the classification performance of the model.

### Performance metrics

In this research, diabetic patient samples with DR were classified as the positive class, while the samples without DR (NDR) were classified as the negative class. The trained machine learning model was employed to make category predictions on the test set, deriving accuracy, precision, F1-measure, sensitivity, and AUC values. Additionally, receiver operating characteristic (ROC) curves were plotted.

### Comprehensive analysis of modelling features and model validation

In our evaluation of models using six metrics (accuracy, precision, recall, F-measure, and area under the curve(AUC) value, it was found that the GBDT model demonstrated outstanding performance across all indicators. By analyzing the features used in developing these models, we determined key correlates associated with the occurrence of DR through voting. We used the selected classifiers to predict and assess DR classification for a subset of data containing these features.

### Interpretability of classifiers SHAP

Machine learning models often have better predictive accuracy than traditional statistical linear models and are well suited to complex real-world behavior. However, they may lack the interpretability of linear models and are often labeled as ‘black box’ models. In this investigation, we used the shapley additive explanations (SHAP)^18,19^ method, proposed by Professor Lloyd Shapley of the University of California, Los Angeles (UCLA). This method provides local explanations for the machine learning model predictions, allowing us to identify non-linear interaction effects. The SHAP method has been valuable in discovering new insights in research related to human health, environmental issues^20^, and socio-economic issues^21^. This method has allowed us to derive significant correlations between modeling features on predicted outcomes and interactions between features. This enabled us to clearly explain the mechanisms by which the important correlates derived in this study affect DR patients.

### Ethics approval and consent to participate

All research methods were conducted in compliance with relevant guidelines and regulations. Ethical approval was secured from the Institutional Review Board (approval number: 2022-MYSZR158). Informed consent was acquired from all participants and/or their legal guardians, and strict confidentiality was maintained throughout the study.

## Results

### Comparison of DR and NDR metrics

Table 1 presents a comparison of DR and NDR metrics within the dataset, showing that a comparison of 85 features between the DR Group and the NDR Group found 61 features to be statistically significant.

**Table 1:** Comparison of baseline variables between the two groups.

Characteristic	0(NDR), N = 1,500	1(DR), N = 1,500	p-value2
AGE	59 (51, 67)	57 (50, 64)	< 0.001
SEX	941 (63%)	933 (62%)	0.763
NATION	71 (4.7%)	89 (5.9%)	0.144
MARITAL_STATUS	1,470 (98%)	1,466 (98%)	0.613
HEIGHT	166 (165, 170)	166 (162, 172)	0.535
WEIGHT	73 (66, 78)	73 (65, 81)	0.539
BP_HIGH	132 (120, 146)	140 (130, 155)	< 0.001
BP_LOW	79 (70, 86)	80 (75, 90)	< 0.001
HEART_RATE	78.0000 (78.0000, 78.0000)	78.0000 (78.0000, 78.0000)	0.982
BMI	26.3 (24.2, 27.7)	26.3 (24.5, 28.1)	0.077
*HYPERTENTION*	953 (64%)	1,093 (73%)	< 0.001
HYPERLIPIDEMIA	407 (27%)	249 (17%)	< 0.001
A_S	791 (53%)	752 (50%)	0.154
*CEREBRAL_APOPLEXTY*	76 (5.1%)	148 (9.9%)	< 0.001
CAROTID_ARTERY_STENOSIS	56 (3.7%)	73 (4.9%)	0.126
FLD	437 (29%)	500 (33%)	0.013
CIRRHOSIS	29 (1.9%)	18 (1.2%)	0.106
CLD	210 (14%)	199 (13%)	0.558
PANCREATIC_DISEASE	26 (1.7%)	22 (1.5%)	0.561
BILIARY_TRACT_DISEASE	197 (13%)	230 (15%)	0.085
NEPHROPATHY	374 (25%)	903 (60%)	< 0.001
RENAL_FALIURE	24 (1.6%)	159 (11%)	< 0.001
NERVOUS_SYSTEM_DISEASE	97 (6.5%)	79 (5.3%)	0.162
CHD	611 (41%)	374 (25%)	< 0.001
MI	123 (8.2%)	67 (4.5%)	< 0.001
CHF	110 (7.3%)	102 (6.8%)	0.569
ARRHYTHMIAS	95 (6.3%)	79 (5.3%)	0.211
RESPIRATORY_SYSTEM_DISEASE	250 (17%)	222 (15%)	0.16
LEADDP	119 (7.9%)	357 (24%)	< 0.001
HEMATONOSIS	117 (7.8%)	327 (22%)	< 0.001
RHEUMATIC_IMMUNITY	66 (4.4%)	37 (2.5%)	0.004
PREGNANT	6 (0.4%)	4 (0.3%)	0.526
ENDOCRINE_DISEASE	398 (27%)	604 (40%)	< 0.001
MEN	65 (4.3%)	43 (2.9%)	0.031
PCOS	2 (0.1%)	1 (< 0.1%)	> 0.999
DIGESTIVE_CARCINOMA	119 (7.9%)	34 (2.3%)	< 0.001
UROLOGIC_NEOPLASMS	22 (1.5%)	9 (0.6%)	0.019
GYNECOLGICAL_TUMOR	69 (4.6%)	30 (2.0%)	< 0.001
BREAST_TUMOR	7 (0.5%)	3 (0.2%)	0.205
LUNG_TUMOR	46 (3.1%)	9 (0.6%)	< 0.001
INTRACRANIAL_TUMOR	11 (0.7%)	5 (0.3%)	0.133
OTHER_TUMOR	173 (12%)	73 (4.9%)	< 0.001
GLU	7.2 (5.9, 9.3)	7.8 (5.8, 10.7)	0.002
GLU_2H	14.93 (14.93, 14.93)	14.93 (14.93, 14.93)	< 0.001
HBA1C	7.00 (6.40, 8.20)	8.00 (6.70, 9.40)	< 0.001
GSP	226 (200, 229)	226 (199, 253)	0.004
TG	1.63 (1.12, 2.27)	1.55 (1.13, 2.33)	0.572
TC	4.43 (3.66, 5.08)	4.53 (3.79, 5.40)	< 0.001
HDL_C	1.04 (0.86, 1.21)	1.02 (0.86, 1.22)	0.65
LDL_C	2.71 (2.05, 3.24)	2.82 (2.21, 3.51)	< 0.001
FBG	3.31 (2.81, 4.07)	3.67 (2.97, 4.75)	< 0.001
UPR_24	1.35 (1.35, 1.35)	1.35 (0.37, 1.47)	0.071
BU	5.4 (4.4, 6.6)	6.4 (5.0, 8.9)	< 0.001
SCR	70 (58, 82)	79 (60, 122)	< 0.001
UCR	5.70 (5.70, 5.70)	5.70 (4.90, 5.70)	< 0.001
SUA	311 (251, 372)	329 (270, 399)	< 0.001
HB	139 (125, 151)	130 (113, 144)	< 0.001
CP	2.20 (2.20, 2.20)	2.20 (1.77, 2.20)	< 0.001
INS	13.8 (13.8, 13.8)	13.8 (9.5, 13.8)	< 0.001
PCV	0.41 (0.37, 0.44)	0.38 (0.33, 0.42)	< 0.001
PLT	210 (174, 251)	211 (173, 254)	0.704
ESR	26 (26, 26)	26 (16, 26)	0.769
TBILI	10.0 (7.6, 13.7)	8.6 (6.0, 12.2)	< 0.001
DBILI	3.20 (2.30, 4.30)	2.30 (1.50, 3.50)	< 0.001
TP	67 (64, 72)	64 (60, 69)	< 0.001
ALB	41.5 (38.7, 44.1)	39.4 (34.8, 42.0)	< 0.001
LDH_L	155 (136, 182)	165 (143, 195)	< 0.001
ALT	21 (14, 31)	16 (12, 24)	< 0.001
AST	17 (14, 23)	16 (13, 20)	< 0.001
GGT	29 (19, 49)	23 (16, 36)	< 0.001
ALP	70 (58, 85)	68 (56, 83)	0.003
LP_A	31.2000 (31.2000, 31.2000)	31.2000 (31.2000, 31.2000)	< 0.001
PL	2.46 (2.46, 2.46)	2.46 (2.46, 2.46)	0.601
PT	13.10 (12.60, 13.60)	12.90 (12.40, 13.50)	< 0.001
PTA	100 (91, 108)	100 (92, 110)	0.028
APTT	35.9 (33.4, 38.9)	36.0 (33.5, 38.7)	0.802
FIBRIN	14.3400 (14.3400, 14.3400)	14.3400 (14.3400, 14.3400)	0.232
ALB_CR	151 (36, 151)	151 (18, 206)	< 0.001
LPS	163 (107, 163)	163 (118, 163)	0.004
CA199	17 (9, 28)	19 (11, 28)	< 0.001
CRP	0.81 (0.32, 1.20)	0.35 (0.31, 1.20)	< 0.001
M1_M2	55.0000 (55.0000, 55.0000)	55.0000 (55.0000, 55.0000)	0.738
TH2	0.4300 (0.4300, 0.4300)	0.4300 (0.4300, 0.4300)	0.592
IBILI	6.8 (5.0, 9.5)	6.2 (4.3, 8.6)	< 0.001
GLO	26.0 (23.0, 29.2)	25.5 (22.4, 28.5)	< 0.001

There were 31 features in the DR Group that were lower than those in the NDR gro-up (P < 0.05), 28 of which were statistically significant, and they were:AGE (Age), MEN (Men), HB (HB), UCR (Urine creatinine), CP (C-peptide), INS (Insulin), PCV (Packed cell volume), and TBILI (Total bilirubin), DBILI(Direct bilirubin), TP (Total protein), ALB (Albumin), ALT (Alanine transaminase), AST (Aspartate transaminase), GGT (Glutamyl transpeptadase), ALP (Alkaline phosphatase), LP_A (Lipoprotein-A), and PT (Prothrombin time), CRP (C-reactive protein), IBILI (Indirectbilirubin), GLO (Globulin), HYPERTENTION (Hypertension), CEREBRAL_APOPLEXTY (Cerebral apoplexty), and FLD (Fatty liver disease), NEPHROPATHY (Nephropathy), RENAL_FALIURE (Renal faliure), LEADDP (Lower extremity arterial disease), HEMATONOSIS (Hematonosis), ENDOCRINE_DISEASE (Endocrine disease), MEN(Men), ALP (Alkaline phosphatase), and FLD (Fatty liver disease) were statistically significant.

There were 30 features in the DR Group that were higher than those in the NDR group (P < 0.05), among which the following 25 features were statistically significant: BP_HIGH (Systolic blood pressure), BP_LOW (Diastolic blood pressure), GLU (Glucose), GLU_2H (2-h postprandial blood glucose), HBA1C (Glycated hemoglobin A1c), GSP (Glycosylated serum protein), TC (Total cholesterol), LDL_C (Low density) lipoprotein cholesterol, FBG (Fibrinogen), BU (Blood urea), SCR (Serum creatinine), SUA (Serum urea), LDH_L (Lactate dehydrogenase), PTA (Prothrombin activity), ALB_CR (Urinary albumin/creatinine ratio), LPS (Lipase), CA199 (Carbohydrate antigen 199), HYPERLIPIDEMIA (Hyperlipidemia), CHD (Coronary heart disease), MI (Myocardial infarction), RHEUMATIC_IMMUNITY (Rheumatic immunity), DIGESTIVE_CARCINOMA (Digestive carcinoma), UROLOGIC_NEOPLASMS (Urologic neoplasms), GYNECOLGICAL_TUMOR (Gynecolgical tumor), LUNG_TUMOR (Lung tumor), OTHER_TUMOR (Other tumor), GLU (Glucose) and GSP (Glycosylated serum pro-tein), LPS (Lipase), RHEUMATIC_IMMUNITY (Rheumatic_immunity), UROLOGIC_NEOPLASMS (Urologic neoplasms) were statistically significant.

### Risk factors

The results from both univariable and multivariable logistic analyses were statistically significant, with *p* < 0.05, as illustrated in Table2. A total of 27 features were identified as correlates affecting DR.

**Table 2:** Univariale and multivarialbe analysis.

Variables	Univariale analysis		Multivarialbe analysis	
	OR (95%CI)	Pvalue	OR (95% CI)	Pvalue
AGE	0.981 (0.974-0.987)	4.5603E-09	**0.982 (0.972**-**0.992)**	**0.00028968**
SEX	0.978 (0.843-1.133)	0.762923909		
NATION	1.27 (0.921-1.749)	0.144		
MARITAL_STATUS	1.136 (0.692-1.867)	0.613		
HEIGHT	0.997 (0.986-1.007)	0.51748		
WEIGHT	1.001 (0.995-1.007)	0.70003		
BP_HIGH	1.018 (1.014-1.021)	5.60E-21	**1.009 (1.003**-**1.015)**	**0.0022348**
BP_LOW	1.022 (1.016-1.028)	7.90E-12	1.002 (0.992-1.013)	0.65854203
HEART_RATE	0.995 (0.983-1.006)	0.35743		
BMI	1.015 (0.994-1.036)	0.172887		
HYPERTENTION	1.541 (1.320-1.800)	4.41E-08	1.028 (0.826-1.279)	0.80525961
HYPERLIPIDEMIA	1.871 (1.567-2.234)	4.44E-12	**0.633 (0.501**-**0.798)**	**0.00011444**
A_S	0.901 (0.781-1.040)	0.154301		
CEREBRAL_APOPLEXTY	2.051 (1.540-2.731)	8.85E-07	**1.575 (1.084**-**2.291)**	**0.01729384**
CAROTID_ARTERY_STENOSIS	0.127 (0.924-1.883)	0.127075		
FLD	0.822 (0.704-0.960)	0.013121	1.208 (0.967-1.508)	0.0962293
CIRRHOSIS	1.623 (0.897-2.936)	0.109105		
CLD	0.940 (0.763-1.158)	0.558406		
PANCREATIC_DISEASE	0.844 (0.476-1.496)	0.560998		
BILIARY_TRACT_DISEASE	1.200 (0.975-1.471)	0.084902		
NEPHROPATHY	4.554 (3.896-5.323)	9.1556E-81	**2.402 (1.907**-**3.025)**	**9.5624E-14**
RENAL_FALIURE	0.137 (0.089-0.212)	3.86E-19	**0.393 (0.230**-**0.672)**	**0.00064969**
NERVOUS_SYSTEM_DISEASE	1.244 (0.916-1.689)	0.162675		
CHD	2.069 (1.771-2.418)	5.99E-20	**1.693 (1.348**-**2.126)**	**5.8334E-06**
MI	1.910 (1.406-2.596)	0.000035	1.006 (0.670-1.511)	0.97714135
CHF	0.922 (0.697-1.219)	0.568797		
ARRHYTHMIAS	1.216 (0.894-1.654)	0.212005		
RESPIRATORY_SYSTEM_DISEASE	1.151 (0.946-1.402)	0.160535		
LEADDP	3.625 (2.904-4.525)	5.21E-30	**0.416 (0.318**-**0.545)**	**1.9321E-10**
HEMATONOSIS	0.303 (0.242-0.380)	2.85E-25	1.193 (0.846-1.682)	0.3139208
RHEUMATIC_IMMUNITY	1.820 (1.209-2.740)	0.004119	0.604 (0.360-1.012)	0.0554894
PREGNANT	1.502 (0.423-5.333)	0.529214		
ENDOCRINE_DISEASE	0.536 (0.459-0.625)	2.18E-15	**1.422 (1.154**-**1.753)**	**0.00094942**
MEN	1.535 (1.037-2.272)	0.032244	**0.437 (0.269**-**0.711)**	**0.00084151**
PCOS	2.001 (0.181-22.095)	0.571227		
DIGESTIVE_CARCINOMA	3.715 (2.520-5.478)	3.42E-11	**0.626 (0.394**-**0.994)**	**0.04709342**
UROLOGIC_NEOPLASMS	2.466 (1.132-5.373)	0.023129	0.561 (0.228-1.380)	0.20799546
GYNECOLGICAL_TUMOR	2.363 (1.530-3.649)	0.000106	**0.498 (0.291**-**0.850)**	**0.01066212**
BREAST_TUMOR	2.340 (0.604-9.065)	0.218696		
LUNG_TUMOR	5.241 (2.556-10.746)	0.000006	**0.356 (0.149**-**0.850)**	**0.0199816**
INTRACRANIAL_TUMOR	2.209 (0.766-6.373)	0.14267		
OTHER_TUMOR	2.548 (1.919-3.384)	1.01E-10	**0.587 (0.404**-**0.854)**	**0.00534148**
GLU	1.033 (1.014-1.053)	0.000612	**0.960 (0.933**-**0.988)**	**0.00488622**
GLU_2H	1.083 (1.046-1.121)	0.000006	**1.057 (1.010**-**1.105)**	**0.01560123**
HBA1C	1.279 (1.223-1.337)	1.22E-27	**1.458 (1.362**-**1.561)**	**1.5551E-27**
GSP	1.001 (1.000-1.002)	0.185663		
TG	1.016 (0.972-1.062)	1.000749		
TC	1.151 (1.090-1.215)	3.49E-07	0.899 (0.780-1.037)	0.14477468
HDL_C	1.224 (0.972-1.541)	0.085889		
LDL_C	1.216 (1.135-1.302)	2.59E-08	1.017 (0.858-1.206)	0.8454485
FBG	1.002 (1.000-1.004)	0.050895		
UPR_24	1.169 (1.083-1.263)	0.000068	**0.809 (0.710**-**0.921)**	**0.00140512**
BU	1.127 (1.103-1.152)	2.67E-27	**1.077 (1.034**-**1.122)**	**0.00041561**
SCR	1.004 (1.003-1.005)	2.87E-18	0.999 (0.997-1.000)	0.09517144
UCR	0.991 (0.966-1.016)	4.84E-01		
SUA	1.002 (1.001-1.003)	1.44E-07	1.000 (0.999-1.001)	0.70566987
HB	0.982 (0.979-0.985)	2.06E-26	**1.038 (1.016**-**1.059)**	**0.00051921**
CP	0.836 (0.759-0.920)	2.53E-04	**0.855 (0.769**-**0.950)**	**0.00367297**
INS	1.002 (0.997-1.006)	4.24E-01		
PCV	0.001 (0.000-0.002)	5.21E-33	**0.000 (0.000**-**0.000)**	**3.3492E-06**
PLT	1.000 (0.999-1.001)	0.938903		
ESR	1.006 (1.002-1.010)	0.004677	**0.993 (0.987**-**0.999)**	**0.02870571**
TBILI	0.952 (0.939-0.965)	1.31E-12	1.686 (0.613-4.639)	0.31191685
DBILI	0.788 (0.754-0.823)	2.52E-26	0.521 (0.189-1.436)	0.20727451
TP	0.930 (0.920-0.941)	8.34E-38	0.979 (0.807-1.189)	0.83054554
ALB	0.911 (0.898-0.923)	1.05E-38	0.966 (0.795-1.174)	0.7273322
LDH_L	1.002 (1.000-1.003)	0.0054	1.000 (0.998-1.002)	0.95422001
ALT	0.980 (0.976-0.985)	8.76E-17	0.995 (0.988-1.003)	0.24167898
AST	0.974 (0.967-0.981)	1.46E-12	0.991 (0.980-1.002)	0.10966714
GGT	0.995 (0.994-0.997)	1.95E-08	0.999 (0.997-1.001)	0.28969922
ALP	0.997 (0.995-0.999)	5.09E-03	0.999 (0.996-1.003)	0.65484853
LP_A	1.003 (0.996-1.009)	4.59E-01		
PL	1.052 (0.811-1.365)	7.00E-01		
PT	0.834 (0.778-0.895)	4.16E-07	**0.823 (0.747**-**0.907)**	**8.5771E-05**
PTA	1.003 (0.999-1.007)	0.173784		
APTT	0.988 (0.977-1.000)	4.29E-02	1.003 (0.987-1.018)	0.74076958
FIBRIN	1.001 (0.979-1.024)	0.930633		
ALB_CR	1.003 (1.002-1.003)	3.57E-21	**1.001 (1.000**-**1.002)**	**0.03234779**
LPS	1.000 (1.000-1.000)	0.872597		
CA199	0.999 (0.998-1.000)	1.80E-01		
CRP	0.902 (0.869-0.937)	1.08E-07	**0.938 (0.892**-**0.985)**	**0.0107959**
M1_M2	1.034 (0.973-1.099)	2.84E-01		
TH2	0.016 (0.000-563.394)	4.41E-01		
IBILI	0.959 (0.942-0.976)	2.00E-06	0.625 (0.227-1.723)	0.36393687
GLO	0.970 (0.956-0.985)	7.90E-05	1.017 (0.837-1.235)	0.86848635

There were 11 significant positive associations with DR, namely: HBA1C (Glycated hemoglobin A1c), NEPHROPATHY (Nephropathy), BP_HIGH(Systolic blood pressure), CEREBRAL_APOPLEXTY(Carotid artery stenosis), CHD (Coronary heart disease), ENDOCRINE_DISEASE(Endocrine disease), GLU_2H(2-h postprandial blood glucose), BU (Blood urea) and ALB_CR(Urinary albumin/creatinine ratio) were consistent with the existing studies^22–29^.

There were 16 negative correlations, which were as follows: AGE (Age), HYPERLIPIDEMIA (Hyperlipidemia), RENAL_FALIURE (Renal faliure), LEADDP (Lower extremity arterial disease), MEN (Men), DIGESTIVE_CARCINOMA (Digestive carcinoma), GYNECOLGICAL_TUMOR (Gynecolgical tumor), LUNG_TUMOR (Lung tumor), OTHER_TUMOR (Other tumor), GLU (Glucose), UPR_24 (Twenty-Four Hours Urinary Protein), CP (C-peptide), and PCV (Packed cell) volume, ESR (Erythrocyte sedimentation rate), Prothrombin time (PT), and CRP (C-reactive protein).Among these factors, CP as a protective factor is consistent with existing studies^29^, while the increase of the remaining 15 factors decreases the risk of DR, which may be caused by some specific circumstances, sample selection bias or other unknown confounding factors, requiring further verification and research.

### Feature selection analysis

**Fig. 2:**
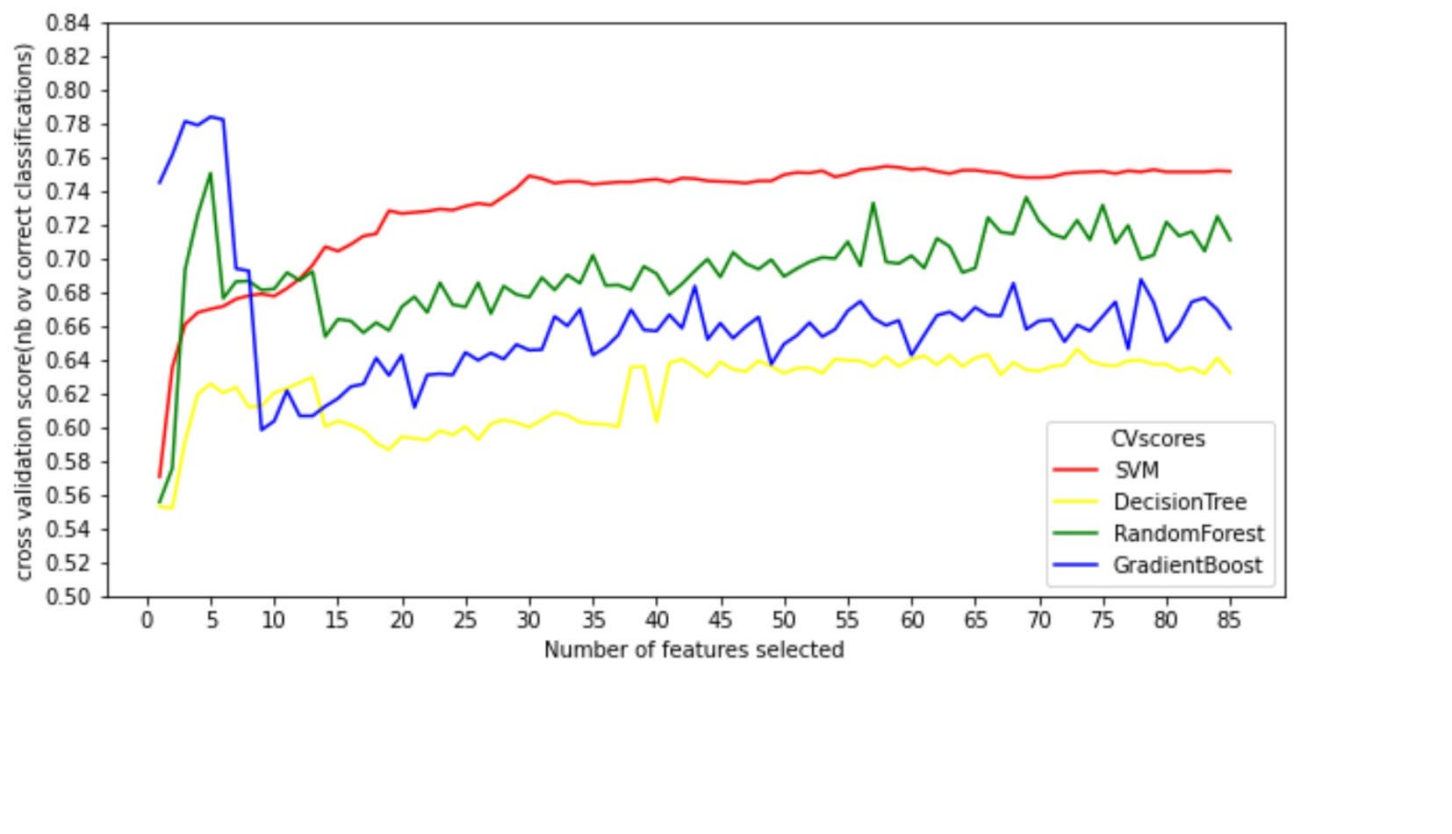
Feature selection cross-verifies the score curve.

An iterative method revealed that retaining a specific number of features during a 3-fold cross-validation (CV) with RFECV improves the predictive performance of the model. The CV scores for four models, each with a different number of features, are provided in Supplementary Table 2, and Fig. 2. The optimal number of features for modeling corresponds to the highest score, which can be used to derive the most relevant features. Each machine learning model utilizes these optimal features (i.e., features with an importance rating of 1, as illustrated in Supplementary Fig. 1) to create a new data subset.

### Hyperparameters

We utilized the GridSearch CV and cross-validation features from the model selection module of Sklearn to assess all possible combinations of discrete hyperparameters in LR, SVM, DT, RF, and GBDT models. This method enabled us to identify the hyperparameter set yielding the highest cross-validation accuracy, which is the subset delivering the best performance (Supplementary Table 3).

### Comparison of prediction model performance

We utilized the four trained machine learning models and LR model to make category prediction on the test set, and obtained the correct number of positive samples (TP), incorrect number of negative samples (FN), incorrect number of positive samples (FP) and correct number of negative samples (TN) as shown in Table 3 according to the prediction results.

**Table 3:** Confusion matrix.

Model	TN	FP	FN	TP
LR	222	55	81	242
SVM	219	74	87	220
DT	233	60	100	207
RF	235	58	68	239
GBDT	249	44	77	230

**Fig. 3:**
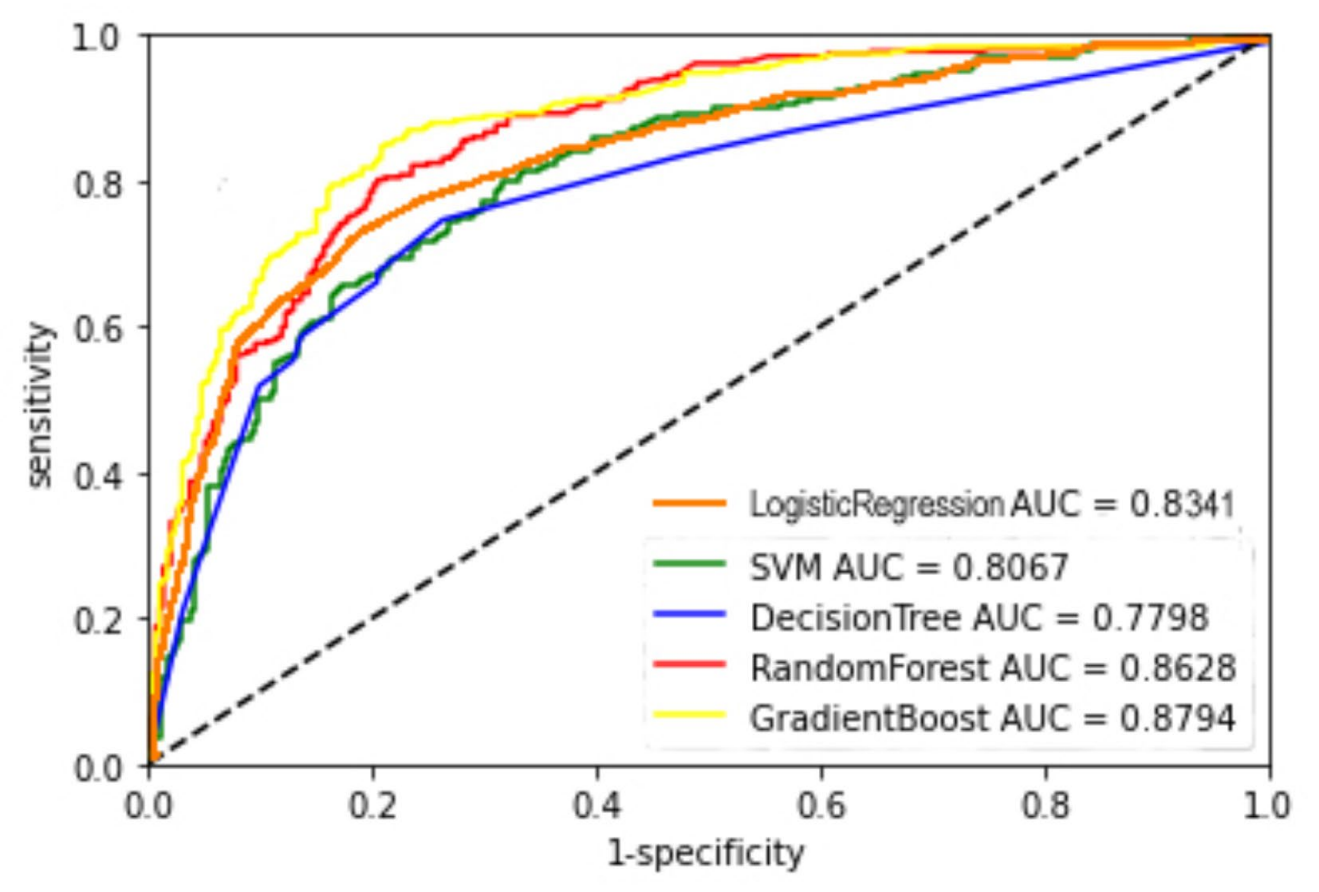
ROC curve.

Key evaluation metrics for each model, including accuracy, precision, sensitivity, F1 value, and AUC value, were generated from testing on the test set (Table 4). The RF and GBDT models outperformed the SVM, DT and LR models in predictive accuracy, with the GBDT model demonstrating the highest accuracy, precision, F1-measure, and AUC value. Figure 3 illustrates the ROC curves for all models, indicating that our optimized machine-learning models hold practical value as the areas under the ROC curves for all models exceed 0.75.

**Table 4:** Model performance indicators.

Model	Accuracy	Precision	Sensitivity	F1-measure	AUC
LR	0.7733	0.8148	0.7492	0.7806	0.8341
SVM	0.7317	0.7483	0.7166	0.7321	0.8067
DT	0.7333	0.7752	0.6743	0.7212	0.7798
RF	0.7900	0.8047	0.7785	0.7914	0.8628
GBDT	0.7983	0.8394	0.7492	0.7917	0.8794

### Comprehensive feature selection and model validation

Supplementary Figs. 1 and 2 depict that among the 85 features analyzed, glycosylated hemoglobin (HBA1C), twenty-four hours urinary protein (UPR_24), serum creatinine (SCR), urinary albumin/creatinine ratio (ALB_CR), blood urea (BU), and c-peptide (CP) are considered critical and relevant factors for DR. A GBDT model was used to analyze the data subset characterized by these factors, resulting in an accuracy of 0.7883 and an AUC of 0.8672. The logistic model, resulting in an accuracy of 0.7733, yielded an AUC value of 0.8341.

### SHAP analysis results

#### Impact of each significant correlate on DR prediction

**Fig. 4:**
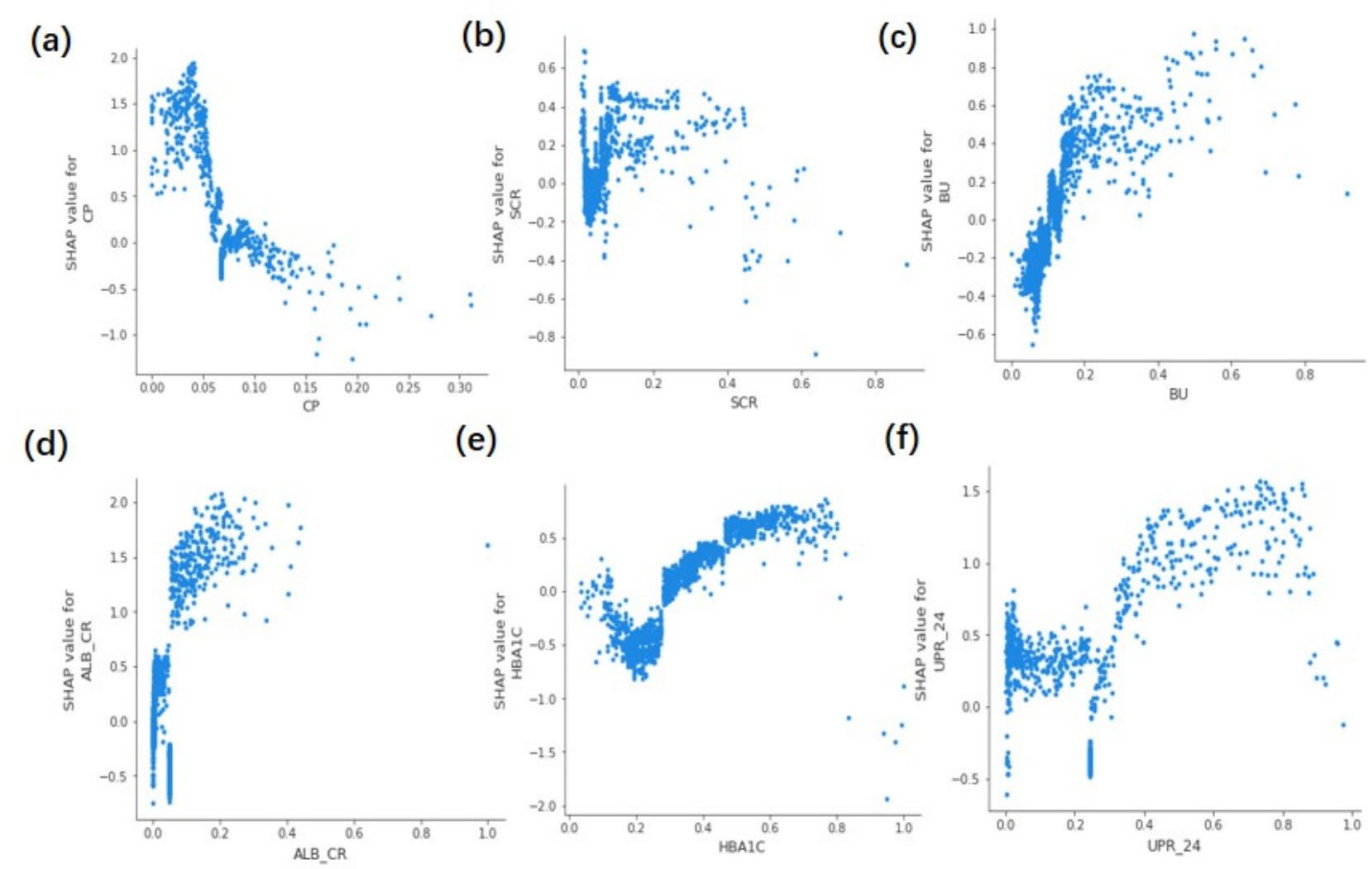
Scatter dependence graph of six important factors related to the occurrence of DR. (**a**) SHAP dependence plot of CP. (**b**) SHAP dependence plot of SCR. (**c**) SHAP dependence plot of BU. (**d**) SHAP dependence plot of ALB_CR. (**e**) SHAP dependence plot of HBAIC. (**f**) SHAP dependence plot of UPR_24.

The SHAP swarm plot (Supplementary Fig. 3a) and violin plot (Supplementary Fig. 3b) provide a comprehensive understanding of how each correlate influences the prediction results. The bar chart displaying the absolute values of SHAP values (Supplementary Fig. 3c) demonstrates the impact of six correlates on the predictive performance of the DR classifier. SHAP dependency plots are utilized to illustrate the effect of each feature on the prediction results (Fig. 4).

#### Explanation of feature interactions

**Fig. 5:**
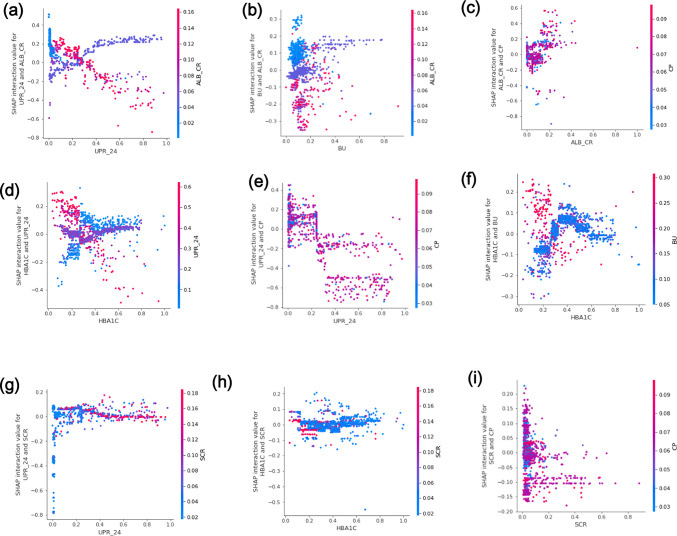
SHAP interaction plot of Pairwise factor.

The SHAP interaction diagram reveals the impact of interactions between two features on DR prediction (Supplementary Fig. 4). A more detailed analysis of the combined impact of these features on DR prediction is presented in Fig. 5.

## Discussion

### Key findings

#### Comparison of LR model and machine learning models in the analysis of DR related factors

Based on four machine learning models, we identified six key factors related to DR. They were ALB_CR (Urinary albumin/creatinine ratio), UPR_24 (Twenty-Four Hours Urinary Protein), CP (C-peptide), HBA1C (Glycated hemoglobin A1c), BU(Blood urea), and SCR (Serum creatinine). The LR model found 27 related factors, These are HBA1C (Glycated hemoglobin A1c), NEPHROPATHY (Nephropathy), and BP_HIGH (Systolic blood pressure), CEREBRAL_APOPLEXTY (Carotid artery stenosis), CHD(Coronary heart disease), ENDOCRINE_DISEASE(Endocrine disease), GLU_2H (2-h postprandial blood glucose), BU(Blood urea), HB(Hemoglobin), ALB_CR(Urinary albumin/creatinine ratio), AGE (Age), HYPERLIPIDEMIA (Hyperlipidemia), RENAL_FALIURE (Renal faliure), LEADDP (Lower extremity arterial disease), MEN(Men), DIGESTIVE_CARCINOMA (Digestive carcinoma), GYNECOLGICAL_TUMOR (Gynecolgical tumor), LUNG_TUMOR (Lung tumor), OTHER_TUMOR (Other tumor), GLU (Glucose), UPR_24 (Twenty-Four Hours Urinary Protein), CP (C-peptide), and PCV (Packed cell) volume, ESR (Erythrocyte sedimentation rate), PT (Prothrombin time), CRP (C-reactive protein), among the 27 relevant factors, five factors were found by machine learning models.

From the above results, we can find that machine learning models such as GBDT can automatically learn from a large number of features and identify the most important factors, which is easier for doctors or researchers to pay attention to and interpret. The LR model identified 27 disease-related factors, which may contain some redundant or less important factors, failing to identify the most critical factors as accurately as the GBDT, but providing a more comprehensive picture of disease-related factors.

Although machine learning models such as GBDT are often better than LR statistical models in terms of prediction performance and can identify key factors, the internal working mechanism of the models is complex and not as intuitive and easy to understand as LR models. This paper adopts SHAP feature importance analysis method to understand which features contribute more to the prediction results and how they affect the prediction results. The interpretability of GBDT is increased.

#### Significant contributions in model development

Presently, there are two main categories of machine learning-based DR prediction models. The first category of DR prediction models is constructed on the foundation of fundus images. For instance, a study conducted by Indian researchers proposed a multi-path convolutional neural network (CNN) and a machine learning classifier for DR to analyze 36,769 fundus images for model development and validation. Post-feature extraction using the M-CNN approach, machine learning classifiers such as SVM, RF, and DT were employed to classify the images into various categories, achieving a DR prediction accuracy exceeding 90%^30^. Casanova et al*.* utilized graded fundus photography and systematic data from 3,443 participants in the ACCORD-Eye study, employing double cross-validation to estimate RF and logistic regression, resulting in a DR prediction accuracy of 75%^31^. Despite their high performance, these models are mainly used in a few developed countries due to the requirement of specialist ophthalmologists and costly medical equipment^32^. Numerous developing countries are currently unable to utilize these models for DR screening.

The second category of DR prediction models relies on physiological and biochemical indicators to determine characteristic values. These models primarily use demographic data, medical history, and test results. Most published studies utilize SVM, artificial neural networks (ANN), RF, logistic regression, and decision trees for DR predictive classification. For example, Tsao et al*.* compared the performance of four machine learning algorithms using 10 features on 536 diabetic patients, employing a fivefold cross-validation. Their findings indicated that SVM achieved 79.5% accuracy in DR classification, outperforming decision trees, artificial neural networks, and logistic regression^33^. Yao et al*.* used 530 Chinese residents (including 423 type 2 diabetic patients) as their study population and utilized univariable and multivariable logistic regression (MLR) to analyze the correlation between DR and biochemical metabolic parameters. Based on the MLR results, they developed a Back Propagation Artificial Neural Network (BP-ANN) model to classify the 423 patients, revealing that the AUC values of the BP-ANN model surpassed those of the MLR model (0.84 vs. 0.77)^34^.

A recent investigation, using data from the Korean National Health and Nutrition Examination Survey (KNHANESV-1 and KNHANESV-2 databases), compared learning models like ridge, elastic net, and LASSO with conventional DR indicators. The LASSO-based sparse learning model demonstrated an AUC of 0.82 and an accuracy of 75.2%, proving effective in predicting DR. Furthermore, Blighe et al*.* undertook an environment-wide association study using NHANES data, analyzing over 400 laboratory parameters linked to DR for predictive purposes. They employed parallel univariable regression models, principal component analysis (PCA), penalized regression, and Random Forest for the selection of independent variables (features). The RF model outperformed the others, with an AUC of 0.84^35^.

In comparison to other studies, our research has shown several improvements. First, given the large number of features in the dataset, we employed recursive feature elimination for iterative model construction. This method eliminates irrelevant and redundant dimensions from the 85 features, retaining only those that are beneficial for learning classification. This approach addresses the issues of overfitting, memory consumption, and time overhead of the model while avoiding the subjectivity, inaccuracy, and unstable results that might be introduced by traditional dimensionality reduction approaches. Second, we explored the performance of four machine learning classification models with hyperparameter optimization and selectively constructed the GBDT model. This model showcases high adaptability across various data types and outperforms other data mining techniques in medical tasks for classification prediction. The application of GBDT in DR classification has not been documented in existing literature. Third, when compared to the classical logistic regression model, our model successfully identified six correlated factors for DR with an AUC value of 0.8672. Conversely, the logistic regression model identified 28 correlated factors but achieved a lower AUC value of 0.8341. This demonstrates the superiority of our model in terms of both the number of correlated factors and the classification prediction accuracy.

#### Examination of influential factors

This research identified ALB_CR (Urinary albumin/creatinine ratio), UPR_24 (Twenty-Four Hours Urinary Protein), BU (Blood urea), HBA1C (Glycated hemoglobin A1c), SCR (Serum creatinine), and CP (C-peptide) as significant correlates of DR, with six pairs of factors demonstrating some interactive effect on the prevalence of DR.

ALB_CR and UPR_24 are commonly used to measure micro-urinary protein levels. The presence of proteinuria, an essential marker of damage to the vascular endothelial system, often indicates widespread microangiopathy in the body^36^. Studies by Li Rui and Li Meifang suggested that diabetic patients with proteinuria had a higher incidence of DR compared to those without proteinuria. They reported a relative risk of 2.638 for the group with microproteinuria (i.e., higher ALB_CR, UPR_24) and 2.702 for the group with substantial albuminuria, thus concluding that microprotein was closely associated with the development of DR^37,38^.

Research conducted by Huang Shufang, Ai Wei, and Fan Ruilei suggests that increased levels of micro-urinary protein indicate an independent risk factor for DR. They proposed that testing for micro-urinary protein can serve as a predictive tool for the progression of DR, enabling early detection of diabetic microangiopathy and reducing the incidence of DR through timely clinical intervention^39–41^. The findings of this study are consistent with the SHAP fovea and dependency plots, where elevated ALB_CR and UPR_24 were significantly associated with DR. Furthermore, a notable rise in the incidence of DR was observed with increased ALB_CR levels compared to UPR_24.

Serum creatinine (SCR) is a byproduct of meat consumption and muscle tissue metabolism. Variations in SCR concentrations are primarily determined by the glomerular filtration rate (GFR) and the filtering capacity of the kidneys. Elevated SCR levels often signify kidney damage, although they are insensitive indicators of kidney parenchymal damage. Extensive renal damage affecting more than half of the kidney can lead to an increase in SCR. However, SCR levels do not indicate an early or mild decline in renal function. Conversely, our research revealed a significantly higher risk of DR development when SCR levels exceeded 0.02 (60 μM/L before data pre-processing), even within the normal value range (44‒133 μM/L). Therefore, monitoring SCR within the normal range can aid in the early detection of DR rather than raising concerns only when SCR levels exceed normal values. Studies have demonstrated a statistically significant difference in SCR levels between DR and NDR groups (*p* < 0.05)^42,43^, proving SCR as an independent risk factor for DR. Individuals with SCR levels exceeding 133 mM/L are 2.006 times more likely to develop DR than those with normal SCR^44^.

Blood urea (BU) is the primary end product of protein metabolism and is removed from the body through glomerular filtration. Research conducted by Wang Yangzhong, Liu Hongfang, and Ma Yue has revealed that BU levels were significantly higher in the DR group compared to the non-DR group, demonstrating a statistically significant difference (*p* < 0.05). BU levels in type 2 diabetic patients were associated with DR development^45^. The SHAP plot results from our study depicted that as BU levels increased, there was a shift from suppression to promotion in the model, predicting a positive result. When the BU index exceeded 0.15 (5 mM/L before data pre-processing), the proportion of DR patients significantly increased, corroborating with the findings of Song Yanan *et al*^46^.

UPR_24, ALB_CR, SCR, and BU are key renal function indicators closely associated with DR risk. This might be attributed to the similar characteristics between the kidney and retina, such as their origin, development, capillary network structure, and filtration barrier function^47^. DR and renal disease may share multiple pathogenic mechanisms. DR development could be influenced by activation of the renin-angiotensin system, impairment of renal function, and genetic, hemodynamic, and lipid metabolism^48^. Moreover, pathogenesis may involve the accumulation of glycosylation end products, activation of the polyol pathway, oxidative stress, growth factors, endoplasmic reticulum stress, inflammatory mediators, and complement activation^49^.

HBA1C reflects not only blood glucose control over time but also plays a role in DR onset and progression. Chronic high blood glucose levels are the primary DR development catalyst. In vivo and in vitro studies have demonstrated that high glucose levels induce pericyte apoptosis^50^. Pericytes provide structural support to capillaries; their loss can lead to localized bulging of capillary walls, contributing to microaneurysm formation, which is the earliest DR clinical sign^51^. Poor glycemic control may increase DR risk. Higher levels of HbA1c in diabetic patients can exacerbate vessel wall damage and capillary occlusion through increased aggregation of erythrocytes, potentially causing tissue ischemia and hypoxia, which can trigger retinal metabolic disorders^52^. Our analysis of the SHAP plot data revealed a strong correlation between HBA1C and DR. The DR prevalence risk increased when HBA1C exceeded 7% and also dropped below 6%. As HBA1C values decreased, the resistance to DR prevalence decreased as well, thus elevating the likelihood of DR prevalence, consistent with previous studies^53^. However, this does not imply that lower glycosylated hemoglobin is always beneficial for diabetic patients. Glycosylated hemoglobin levels maintained below 6% have been associated with increased hypoglycemic episodes and higher mortality. Therefore, it is essential to maintain control over glycosylated hemoglobin. Some scholars recommend maintaining glycosylated hemoglobin levels between 6.5% and 7% for optimal management^54^.

C-peptide (CP) serves as an indicator of the body’s insulin secretion level and is a reliable gauge of the reserve function of pancreatic islet cells. Numerous domestic and international studies have revealed that CP, an active hormone, specifically binds to endothelial cells in a stereotactic manner. In a high-glucose environment, it stimulates Na^+^-K^+^-ATPase on the cell membrane surface of endothelial cells, inhibits nuclear factor-kb, and activates endothelial-type nitric oxide synthase, leading to improved blood flow and vascular permeability within the retinal vasculature^55^. Furthermore, in diabetic patients, C-peptide activates AMP-activated protein kinase alpha to inhibit intracellular reactive oxygen species-mediated endothelial apoptosis, thereby improving endothelial dysfunction^56^. A study by Bo et al*.* observed that the lowest fasting CP levels in type 2 diabetic patients correlated with the highest incidence of DR at baseline and at follow-up, while the risk of retinopathy in diabetic patients negatively correlated with the highest fasting CP levels^55^. A study by Wang et al*.* (2018) categorized four different groups (Q1, Q2, Q3, and Q4) based on fasting CP levels^57^. The results indicated a progressive decrease in the prevalence of DR as fasting CP levels increased. The results of our SHAP plot also confirmed CP as a protective factor against DR. A lower CP value correlated with a higher likelihood of DR, whereas an increase in CP value significantly decreased the likelihood of DR.

The SHAP plot results in this study revealed a significant potential interaction between six pairs of characteristics ‒ UPR_24 and ALB_CR, BU and ALB_CR, CP and ALB_CR, HBA1C and UPR_24, CP and UPR_24, and HBA1C and BU ‒ on the prevalence of DR. Particularly, the interaction between UPR_24 and ALB_CR became evident at lower ALB_CR values. The risk of developing DR increased with increasing UPR_24 levels. BU exhibited a partial interaction with ALB_CR, with changes in BU non-significantly affecting the predicted outcome, but diabetic patients with lower ALB_CR values had a higher risk of developing DR. Moreover, CP displayed interactions with ALB_CR in patients with both higher and lower CP values, influencing the risk of DR. The difference in DR risk linked to UPR_24 was dependent on HBA1C levels, with higher HBA1C levels increasing the risk of DR at lower UPR_24 values. In the interaction between HBA1C and BU, the probability of DR was increased when the HBA1C value was below 7% and the BU value was higher. Conversely, if the HBA1C value exceeded 7%, the likelihood of DR increased even with a lower BU value. These interactions suggest that paying careful attention to one indicator while testing another could provide a more comprehensive prediction of DR. Furthermore, this conclusion offers a new research direction for the pathological mechanism of DR in patients who maintain good control of one indicator.

### Limitations and outlook

Despite the improvements and novel findings in this study, several limitations persist: (1) Our study used a dataset of 3000 records, which is a relatively small sample size, limited to only Chinese patients. It is recommended that the scope of the study be broadened to include diabetic populations from multiple countries, thus allowing our model to predict and diagnose DR more comprehensively. (2) Numerous fields in the sample had missing data, and filling them with mean values introduces a certain level of error, consequently affecting the predictive performance of our model. Future research should consider using cluster interpolation and model interpolation to reduce this error and select a dataset with fewer missing values. (3) The duration of the disease was not included in our data, which has a significant correlation with DR. Hence, future data collection should include the duration of patients’ diseases^58,59^. (4) The understanding of the impact of the interaction of two characteristics on DR prediction remains in the exploratory stage, with limited literature supporting most of these interactions. Future research should further investigate these potential interactions and their implications for DR prediction and management.

## Electronic supplementary material

Below is the link to the electronic supplementary material.

Supplementary Material 1

## Data Availability

The data supporting the findings of this study are freely available from the National Population Health Sciences Data Center data warehousing PHDA. Its website is https://www.ncmi.cn//phda/dataDetails.do?id=CSTR:A0006.11.A0005.201905.000282.
