# Supplementary material for: An enhanced machine learning algorithm for type 2 diabetes prognosis with a detailed examination of Key correlates
Source: Sci Rep. 2024 Nov 1;14:26355. doi: 10.1038/s41598-024-75898-w (PMC11530678; doi:10.1038/s41598-024-75898-w)
Supplement: Supplementary file 1 — Supplementary Material 1 [file 41598_2024_75898_MOESM1_ESM.docx]

| **Supplementary Table 1-4 and Supplementary Fig.1-4** | | | | | | | |
| --- | --- | --- | --- | --- | --- | --- | --- |
| **Supplementary Table 1 Information of variables .** | | | | | | | |
| **No** | **Features** | **Type** | **Description** | **No** | **Features** | **Type** | **Description** |
| 1 | AGE | Numeric | Age | 44 | GLU_2H | Numeric | 2-hour postprandial blood glucose |
| 2 | SEX | Boolean | Sex | 45 | HBA1C | Numeric | Glycated hemoglobin A1c |
| 3 | NATION | Boolean | Nation | 46 | GSP | Numeric | Glycosylated serum protein |
| 4 | MARITAL_STATUS | Boolean | Marital status | 47 | TG | Numeric | Triglyceride |
| 5 | HEIGHT | Numeric | Height | 48 | TC | Numeric | Total cholesterol |
| 6 | WEIGHT | Numeric | Weight | 49 | HDL_C | Numeric | High-density lipoprotein cholesterol |
| 7 | BP_HIGH | Numeric | Systolic blood pressure | 50 | LDL_C | Numeric | Low-density lipoprotein cholesterol |
| 8 | BP_LOW | Numeric | Diastolic blood pressure | 51 | FBG | Numeric | Fibrinogen |
| 9 | HEART_RATE | Numeric | Heat rate | 52 | UPR_24 | Numeric | Twenty-Four Hours Urinary Protein |
| 10 | BMI | Numeric | BMI (kg/m^2^  ) = weight (kg)/height^2^ (m) | 53 | BUN | Numeric | Blood urea nitrogen |
| 11 | HYPERTENSION | Boolean | Hypertension | 54 | BU | Numeric | Blood urea |
| 12 | HYPERLIPIDEMIA | Boolean | Hyperlipidemia | 55 | SCR | Numeric | Serum creatinine |
| 13 | A_S | Boolean | Atherosclerosis | 56 | UCR | Numeric | [Urine creatinine](https://www.so.com/link?m=zGzspu8g4dvZdspAYBwWH83wMtsJMM0kf3GMZqOqFT+yF7Q4FcSSoPJ5PUlCjtdVq/AmRLlp67HiXWGoACR74Km36++1kFBTA3T/qcyBP2l7qqBZg/29CtPwOE+Althy8ShBle9/J3A5dA+PhdnEF/6vaxhZhfZwvA+tNbCmM5yyuaJK25WHHaxD6x2lCjRqFDMnpMGe/yGC21iFTpvryHXlviAIS3WH2/ZqDrZVNPVDv7D2sbHusS9DtcczyrxDnCBrbAm++k9rC+5H5dXla17b9mk1OBRrZ) |
| 14 | CEREBRAL_APOPLEXTY | Boolean | Cerebral apoplexty | 57 | SUA | Numeric | Serum urea |
| 15 | CAROTID_ARTERY_STENOSIS | Boolean | Carotid artery stenosis | 58 | HB | Numeric | Hemoglobin |
| 16 | FLD | Boolean | Fatty liver disease | 59 | CP | Numeric | C-peptide |
| 17 | CIRRHOSIS | Boolean | Cirrhosis | 60 | INS | Numeric | Insulin |
| 18 | CLD | Boolean | Other chronic liver diseases | 61 | PCV | Numeric | Packed cell volume |
| 19 | PANCREATIC_DISEASE | Boolean | Pancreatic disease | 62 | PLT | Numeric | Blood platelet |
| 20 | BILIARY_TRACT_DISEASE | Boolean | Biliary tract disease | 63 | ESR | Numeric | Erythrocyte sedimentation rate |
| 21 | NEPHROPATHY | Boolean | Nephropathy | 64 | TBILI | Numeric | Total bilirubin |
| 22 | RENAL_FALIURE | Boolean | Renal faliure | 65 | DBILI | Numeric | Direct bilirubin |
| 23 | NERVOUS_SYSTEM_DISEASE | Boolean | Nervous system disease | 66 | TP | Numeric | Total protein |
| 24 | CHD | Boolean | Coronary heart disease | 67 | ALB | Numeric | Albumin |
| 25 | MI | Boolean | Myocardial infarction | 68 | LDH_L | Numeric | Lactate dehydrogenase |
| 26 | CHF | Boolean | Chronic heart failure | 69 | ALT | Numeric | Alanine transaminase |
| 27 | ARRHYTHMIAS | Boolean | Arrhythmias | 70 | AST | Numeric | Aspartate transaminase |
| 28 | RESPIRATORY_SYSTEM_DISEASE | Boolean | Respiratory system diseases | 71 | GGT | Numeric | Glutamyl transpeptadase |
| 29 | LEADDP | Boolean | Lower extremity arterial disease | 72 | ALP | Numeric | Alkaline phosphatase |
| 30 | HEMATONOSIS | Boolean | Hematonosis | 73 | LP_A | Numeric | Lipoprotein-A |
| 31 | RHEUMATIC_IMMUNITY | Boolean | Rheumatic immunity | 74 | PL | Numeric | Phospholipid |
| 32 | PREGNANT | Boolean | Pregnant | 75 | PT | Numeric | Prothrombin time |
| 33 | ENDOCRINE_DISEASE | Boolean | Endocrine disease | 76 | PTA | Numeric | Prothrombin activity |
| 34 | MEN | Boolean | Men | 77 | APTT | Numeric | active partial thromboplastin time |
| 35 | PCOS | Boolean | Polycystic ovary syndrome | 78 | FIBRIN | Numeric | Fibrin |
| 36 | DIGESTIVE_CARCINOMA | Boolean | Digestive carcinoma | 79 | ALB_CR | Numeric | Urinary albumin/creatinine ratio |
| 37 | UROLOGIC_NEOPLASMS | Boolean | Urologic neoplasms | 80 | LPS | Numeric | Lipase |
| 38 | GYNECOLGICAL_TUMOR | Boolean | Gynecolgical tumor | 81 | CA199 | Numeric | Carbohydrate antigen 199 |
| 39 | BREAST_TUMOR | Boolean | Breast tumor | 82 | CRP | Numeric | C-reactive protein |
| 40 | LUNG_TUMOR | Boolean | Lung tumor | 83 | M1_M2 | Numeric | M1 macrophages and M2 macrophages |
| 41 | INTRACRANIAL_TUMOR | Boolean | Intracranial tumor | 84 | TH2 | Numeric | T helper 2 cell |
| 42 | OTHER_TUMOR | Boolean | Other tumor | 85 | IBILI | Numeric | Indirect bilirubin |
| 43 | GLU | Numeric | Glucose | 86 | GLO | Numeric | Globulin |

**Supplementary Table 2** Number of features and cross validation score

| Number Of Features | SVM | DT | RandomForest | GradientBoost |
| --- | --- | --- | --- | --- |
| 1 | 0.57066667 | 0.553 | 0.55566667 | 0.745 |
| 2 | 0.63533333 | 0.552 | 0.57566667 | 0.76133333 |
| 3 | 0.661 | 0.59166667 | 0.69333333 | 0.78133333 |
| 4 | 0.668 | 0.61966667 | 0.726 | 0.779 |
| 5 | 0.67 | 0.62566667 | **0.75066667** | **0.784** |
| 6 | 0.67166667 | 0.62033333 | 0.67633333 | 0.78233333 |
| 7 | 0.676 | 0.62366667 | 0.68633333 | 0.694 |
| 8 | 0.678 | 0.612 | 0.68666667 | 0.69266667 |
| 9 | 0.679 | 0.61266667 | 0.68133333 | 0.59833333 |
| 10 | 0.67766667 | 0.62033333 | 0.682 | 0.60366667 |
| 11 | 0.68233333 | 0.623 | 0.69166667 | 0.62166667 |
| 12 | 0.688 | 0.62633333 | 0.687 | 0.60666667 |
| 13 | 0.696 | 0.62966667 | 0.69233333 | 0.60666667 |
| 14 | 0.707 | 0.60033333 | 0.65366667 | 0.61233333 |
| 15 | 0.70433333 | 0.60366667 | 0.664 | 0.617 |
| 16 | 0.70833333 | 0.60133333 | 0.663 | 0.624 |
| 17 | 0.71333333 | 0.598 | 0.656 | 0.62566667 |
| 18 | 0.71466667 | 0.59066667 | 0.662 | 0.641 |
| 19 | 0.72833333 | 0.58666667 | 0.65733333 | 0.63066667 |
| 20 | 0.72666667 | 0.59433333 | 0.67133333 | 0.64266667 |
| 21 | 0.72733333 | 0.59333333 | 0.67733333 | 0.61166667 |
| 22 | 0.728 | 0.59233333 | 0.668 | 0.631 |
| 23 | 0.72933333 | 0.598 | 0.68566667 | 0.63166667 |
| 24 | 0.72866667 | 0.59533333 | 0.67266667 | 0.631 |
| 25 | 0.731 | 0.60033333 | 0.67133333 | 0.64433333 |
| 26 | 0.73266667 | 0.59266667 | 0.68566667 | 0.63966667 |
| 27 | 0.73166667 | 0.602 | 0.66733333 | 0.644 |
| 28 | 0.73666667 | 0.60433333 | 0.68366667 | 0.64033333 |
| 29 | 0.74166667 | 0.60266667 | 0.67866667 | 0.649 |
| 30 | 0.749 | 0.6 | 0.677 | 0.64566667 |
| 31 | 0.74733333 | 0.60433333 | 0.68866667 | 0.646 |
| 32 | 0.74466667 | 0.60866667 | 0.68133333 | 0.66566667 |
| 33 | 0.74566667 | 0.607 | 0.69033333 | 0.66 |
| 34 | 0.74566667 | 0.603 | 0.68533333 | 0.67 |
| 35 | 0.744 | 0.602 | 0.702 | 0.64266667 |
| 36 | 0.74466667 | 0.60166667 | 0.684 | 0.64733333 |
| 37 | 0.74533333 | 0.60033333 | 0.68433333 | 0.65466667 |
| 38 | 0.74533333 | 0.63566667 | 0.68133333 | 0.66966667 |
| 39 | 0.74633333 | 0.636 | 0.69533333 | 0.65766667 |
| 40 | 0.747 | 0.603 | 0.691 | 0.657 |
| 41 | 0.74533333 | 0.638 | 0.67866667 | 0.66666667 |
| 42 | 0.74766667 | 0.64033333 | 0.685 | 0.65866667 |
| 43 | 0.74733333 | 0.63566667 | 0.69266667 | 0.68366667 |
| 44 | 0.746 | 0.63 | 0.69966667 | 0.652 |
| 45 | 0.74566667 | 0.63866667 | 0.689 | 0.66166667 |
| 46 | 0.74533333 | 0.63433333 | 0.70366667 | 0.65266667 |
| 47 | 0.74466667 | 0.633 | 0.697 | 0.65966667 |
| 48 | 0.746 | 0.63933333 | 0.69366667 | 0.66533333 |
| 49 | 0.746 | 0.636 | 0.69933333 | 0.63733333 |
| 50 | 0.74966667 | 0.632 | 0.68933333 | 0.64933333 |
| 51 | 0.751 | 0.635 | 0.694 | 0.65433333 |
| 52 | 0.75066667 | 0.63533333 | 0.698 | 0.662 |
| 53 | 0.752 | 0.632 | 0.70066667 | 0.65366667 |
| 54 | 0.74833333 | 0.64033333 | 0.7 | 0.658 |
| 55 | 0.75 | 0.63966667 | 0.71 | 0.669 |
| 56 | 0.75266667 | 0.63933333 | 0.69566667 | 0.67466667 |
| 57 | 0.75333333 | 0.636 | 0.733 | 0.66466667 |
| 58 | **0.75466667** | 0.642 | 0.698 | 0.66033333 |
| 59 | 0.754 | 0.636 | 0.697 | 0.66333333 |
| 60 | 0.75266667 | 0.64033333 | 0.70166667 | 0.64266667 |
| 61 | 0.75333333 | 0.64233333 | 0.69433333 | 0.65466667 |
| 62 | 0.75166667 | 0.637 | 0.712 | 0.66633333 |
| 63 | 0.75033333 | 0.64266667 | 0.707 | 0.66833333 |
| 64 | 0.75233333 | 0.636 | 0.69166667 | 0.66333333 |
| 65 | 0.75233333 | 0.641 | 0.69433333 | 0.671 |
| 66 | 0.75133333 | 0.643 | 0.72433333 | 0.66633333 |
| 67 | 0.75066667 | 0.631 | 0.71566667 | 0.666 |
| 68 | 0.74866667 | 0.63833333 | 0.71466667 | 0.68533333 |
| 69 | 0.748 | 0.634 | 0.73633333 | 0.658 |
| 70 | 0.748 | 0.63333333 | 0.72233333 | 0.663 |
| 71 | 0.74833333 | 0.636 | 0.71466667 | 0.66366667 |
| 72 | 0.75033333 | 0.637 | 0.712 | 0.65066667 |
| 73 | 0.751 | **0.64633333** | 0.72266667 | 0.66066667 |
| 74 | 0.75133333 | 0.63933333 | 0.711 | 0.657 |
| 75 | 0.75166667 | 0.637 | 0.73166667 | 0.66566667 |
| 76 | 0.75033333 | 0.63633333 | 0.709 | 0.67433333 |
| 77 | 0.752 | 0.63933333 | 0.71966667 | 0.64633333 |
| 78 | 0.75133333 | 0.63966667 | 0.69966667 | 0.68766667 |
| 79 | 0.75266667 | 0.63733333 | 0.702 | 0.674 |
| 80 | 0.75133333 | 0.63733333 | 0.72166667 | 0.65066667 |
| 81 | 0.75133333 | 0.63333333 | 0.71333333 | 0.66 |
| 82 | 0.75133333 | 0.63533333 | 0.716 | 0.67433333 |
| 83 | 0.75133333 | 0.63166667 | 0.70433333 | 0.67666667 |
| 84 | 0.752 | 0.641 | 0.725 | 0.66966667 |
| 85 | 0.75166667 | 0.63233333 | 0.711 | 0.65866667 |


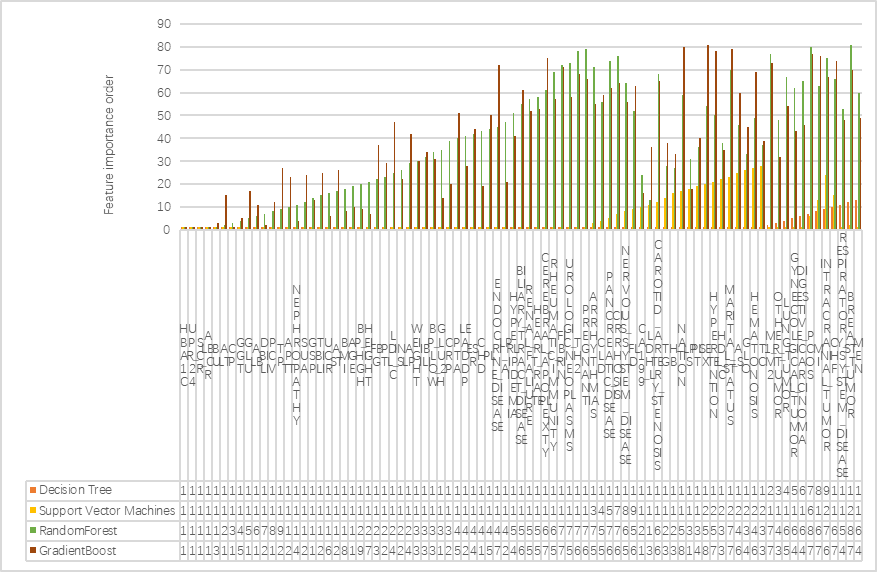


**Supplementary Fig.1 Rank of feature importance**

**Supplementary Table 3** Optimal hyperparameters of the model

| Models | Parameters | Optimized results of grid search |
| --- | --- | --- |
| LR | C | 100 |
|  | penalty | 12 |
| SVM | C | 10.0 |
|  | gamma | 0.1 |
|  | kernel | rbf |
| DT | max_depth | 4 |
|  | min_samples_split | 4 |
| RF | max_features | 5 |
|  | max_depth | 3 |
|  | n_estimators | 40 |
| GBDT | max_features | sqrt |
|  | min_samples_leaf | 2 |
|  | n_estimators | 80 |
|  | subsample | 0.66 |


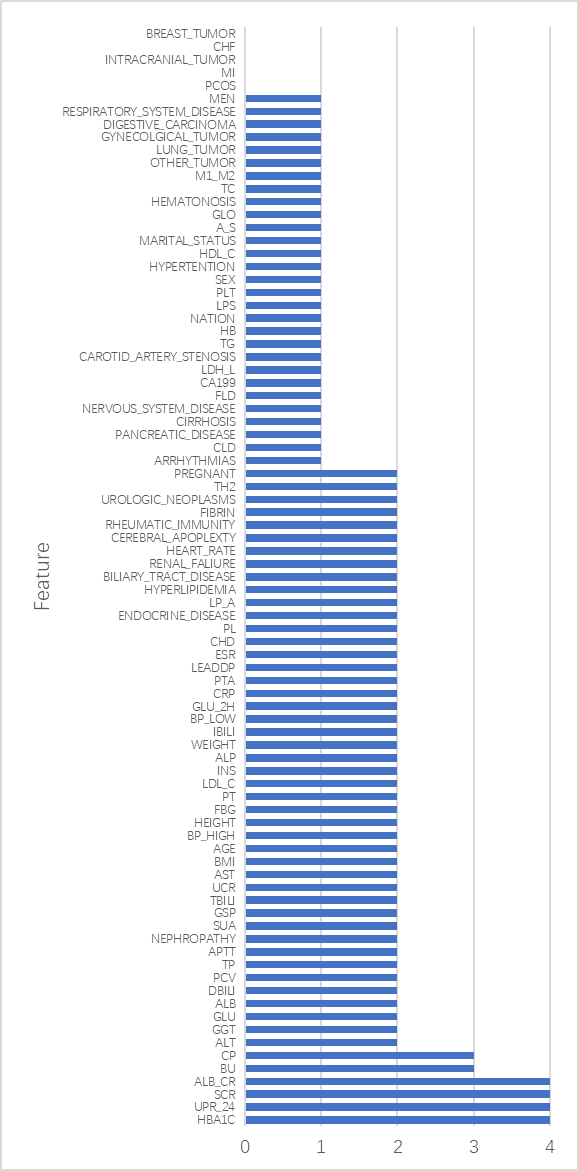


**Supplementary Fig.2** the frequency of all features in the four models with an importance rating of 1

**Supplementary Table 4** Optimal hyperparameters of the model

| Models | Parameters | Optimized results of grid search |
| --- | --- | --- |
| SVM | C | 10.0 |
|  | gamma | 0.1 |
|  | kernel | rbf |
| DT | max_depth | 4 |
|  | min_samples_split | 4 |
| RF | max_features | 5 |
|  | max_depth | 3 |
|  | n_estimators | 40 |
| GBDT | max_features | sqrt |
|  | min_samples_leaf | 2 |
|  | n_estimators | 80 |
|  | subsample | 0.66 |


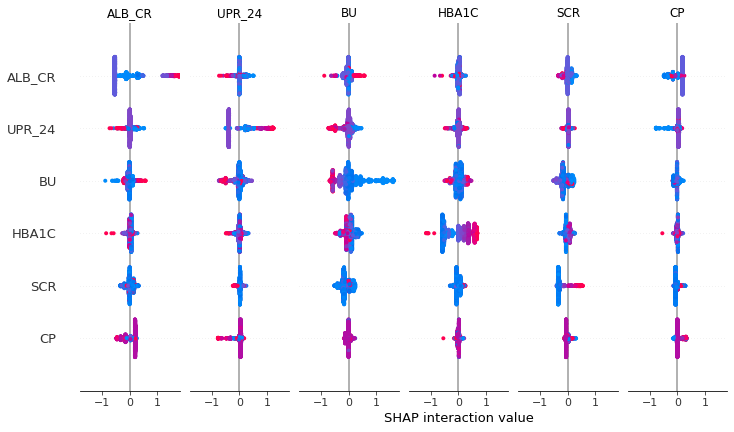


**Supplementary Fig.**4 Summary of the interaction of six important related factors


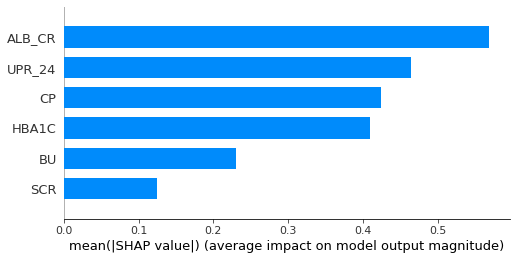

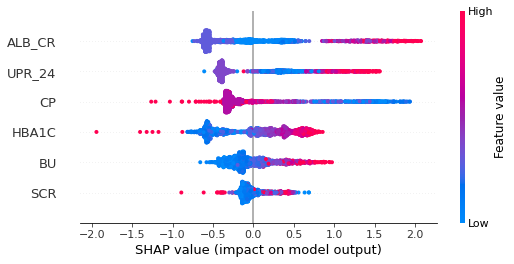

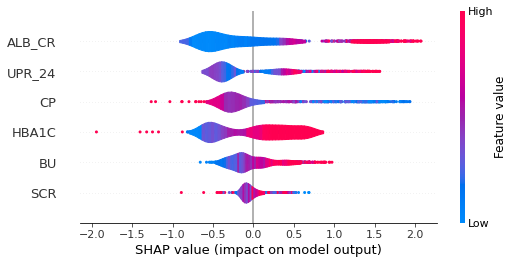


**Supplementary Fig.**3 SHAP graph of important factors related to DR. （a）Map of colonies where DR Occurs. （b）Colony map affecting the occurrence of DR. （c）Bar chart of absolute value of SHAP values for each influencing factor.

**(a)**

**(b)**

**(c)**
